# Of Microbes and Minds: A Narrative Review on the Second Brain Aging

**DOI:** 10.3389/fmed.2018.00053

**Published:** 2018-03-02

**Authors:** Riccardo Calvani, Anna Picca, Maria Rita Lo Monaco, Francesco Landi, Roberto Bernabei, Emanuele Marzetti

**Affiliations:** ^1^Department of Geriatrics, Neurosciences and Orthopedics, Agostino Gemelli University Polyclinic, Catholic University of the Sacred Heart, Rome, Italy

**Keywords:** gut microbiota, neurological disorders, inflamm-aging, gut–brain crosstalk, gut metabolism, brain development, Alzheimer, Parkinson

## Abstract

In recent years, an extensive body of literature focused on the gut–brain axis and the possible role played by the gut microbiota in modulating brain morphology and function from birth to old age. Gut microbiota has been proposed as a relevant player during the early phases of neurodevelopment, with possible long-standing effects in later life. The reduction in gut microbiota diversity has also become one of the hallmarks of aging, and disturbances in its composition are associated with several (age-related) neurological conditions, including depression, Alzheimer’s disease, and Parkinson’s disease. Several pathways have been evoked for gut microbiota–brain communication, including neural connections (vagus nerve), circulating mediators derived by host-bacteria cometabolism, as well as the influence exerted by gut microbiota on host gut function, metabolism, and immune system. Although the most provoking data emerged from animal studies and despite the huge debate around the possible epiphenomenal nature of those findings, the gut microbiota–brain axis still remains a fascinating target to be exploited to attenuate some of the most burdensome consequences of aging.

## Gut Microbiota and Central Nervous System (CNS) in Health and Disease: I “Gut” a Feeling

Over the past decades, few aspects of human physiology have attracted the interest of researchers all over the world as the interaction between gut microbiota and human host (1). According to the current literature, the human holobiont (or superorganism) contains at least the same number of microorganisms (bacteria, archaea, fungi, and viruses) as its own cells (2). More than a billion years of mammalian–microbial coevolution have shaped a life-long interdependency (3). Growing evidence suggests that gut microbiota may be “at the intersection of everything,” being implicated in virtually all physiological or pathological situations (1). Gut microbiota has been implicated in the maturation and modulation of the host immune response (4), interactions (positive and negative) with pathogens (5), regulation of bone density (6), vitamin biosynthesis (7), intestinal 5–10% of daily host energy requirements derives from gut microbiota metabolic activities (8).

Not surprisingly, gut microbiota composition and activities have been associated with a plethora of conditions, ranging from obesity to cardiovascular disease, chronic inflammatory diseases, and cancer (9–11).

**Figure 1:**
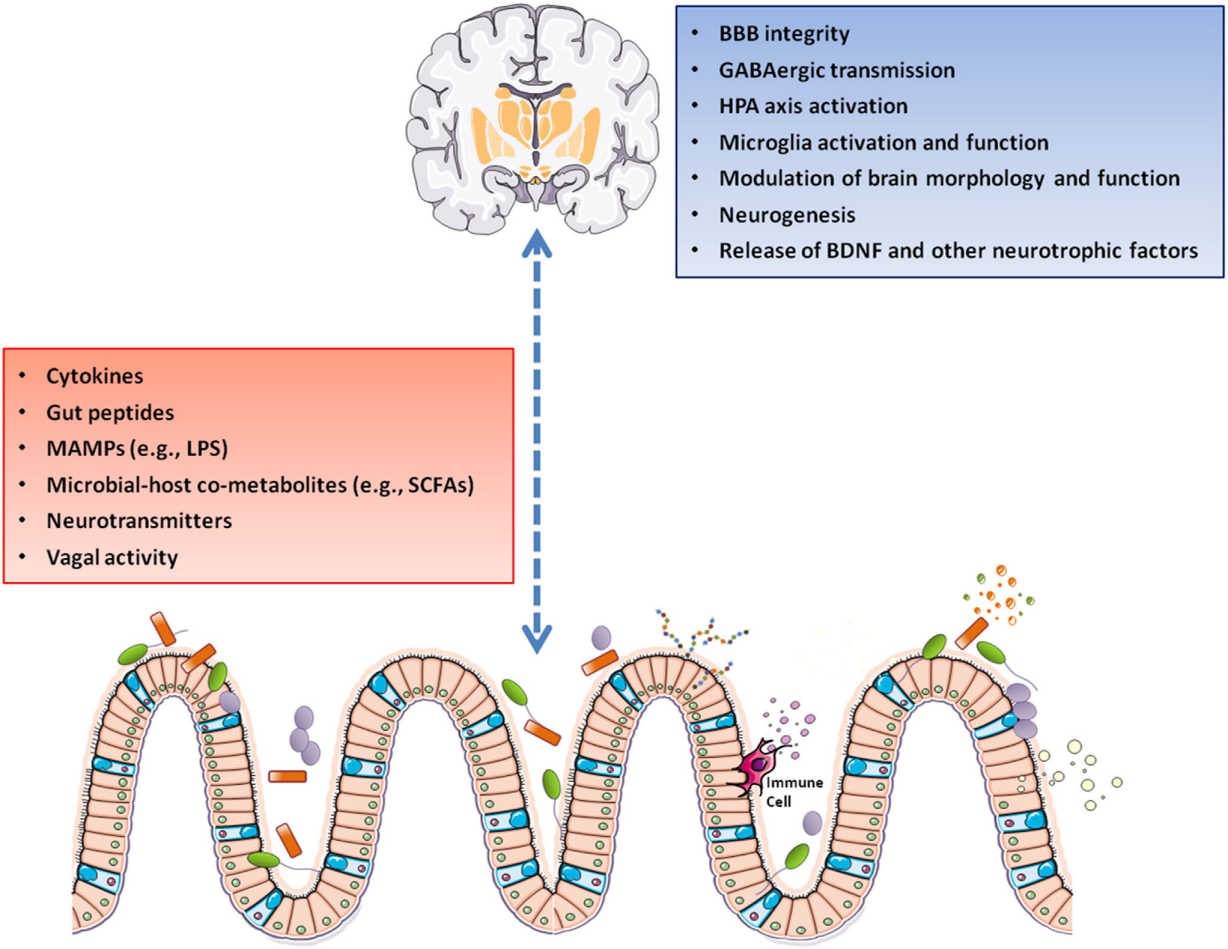
A gut–brain axis supports the interactions between gut microbiota and the CNS through direct and indirect pathways involving vagal nerve activation, cytokine production, and release of neuropeptide/neurotransmitters and SCFAs. These mediators can pass the BBB and control the maturation and activation of brain immune cells (microglia). Following its activation, microglia modulates immune surveillance, synaptic pruning, and clearance of debris. On the other side, the HPA axis can suppress microglia activation, as well as influence cytokine release and trafficking of monocytes from the periphery to the brain. Abbreviations: BBB, blood–brain barrier; BDNF, brain-derived neurotrophic factor; GABA, gamma-aminobutyric acid; HPA axis, hypothalamic–pituitary–adrenal axis; LPS, lipopolysaccharide; MAMPs, microbe-associated molecular patterns; SCFAs, short-chain fatty acids; CNS, central nervous system.

Recently, a great emphasis has been placed on the role of intestinal microbiota in regulating the gut–brain axis (12–15). Gut microbiota and brain may influence one another through several pathways (Figure 1). Gut microbes–brain bidirectional communication is mediated by the vagus nerve that conveys information from the gastrointestinal tract to the CNS and back from CNS to the intestine to modulate intestinal motility, release of neurotransmitters and intestinal immune tone (16, 17). The sympathetic branch of the autonomic nervous system is also involved in intestinal homeostasis and gut immune regulation (18). Gut microbiota may also synthesize (or modulate the synthesis of) a number of neurotransmitters, including dopamine (DA), serotonin (5-HT), noradrenaline (NA), and gamma-aminobutyric acid (19–22). The hypothalamic–pituitary–adrenal axis (HPA axis) is another bidirectional route of communication through which host and gut microbes may interact to orchestrate the core response to both physical and psychological stress challenges (23–25). Bacterial metabolic activities may influence host metabolism and lead to the production of metabolites with neuroactive properties, including short-chain fatty acids (SCFAs) and dietary amino acid catabolites (26, 27). Finally, bacterial mediators in the forms of microbe-associated molecular patterns may drive neuroinflammation (28).

Through all these pathways, gut microbiota exerts a widespread influence on key neurological and behavioral processes and may be involved in critical phases of neurodevelopment and neurodegenerative disorders (12–14, 29). In this scenario, microbial activities on gut–brain axis seem to be especially relevant at the two extremities of human life course (13, 15). Early-life gut microbiota may play a role in shaping neuronal networks influencing cognitive, emotional, and social domains (13). Aging is associated with a reduction in microbial complexity, while alterations in intestinal microbiota composition, structure, and function have been retrieved in older individuals with Alzheimer’s disease (AD) and Parkinson’s disease (PD) (30, 31).

In this narrative review, recent evidence on life-long gut microbiota–brain axis is summarized, with a particular focus on aging and age-related neurodegenerative diseases. All accessible relevant studies written in English were included.

## Gut Microbiota and Neurodevelopment: Early Origins of Late Neurological Diseases?

The notion of “developmental origins of health and disease” poses that prenatal and perinatal life stages are critical periods in which environmental stimuli exert direct and indirect effects on the fetus that might be reflected in later health and disease conditions (32). In this context, early host–microbiota interactions seem to be among the most relevant factors in “programming” adult phenotypes (33). It has been postulated that a succession of microbiota components occurs through major steps at birth (depending on the timing and mode of delivery), then during breastfeeding and first interactions with the environment, and finally during and after weaning. Maternal–host factors (genetic background of mother–infant dyad) and perinatal exposure to antibiotics are among the most relevant factors in shaping the newborn’s microbiota (33, 34). Interaction with colonizing microbiota may prime immune and metabolic functions and have a long-lasting influence on the risk of developing several conditions in later life, including gastrointestinal, allergic, autoimmune, and metabolic diseases (34).

Neurodevelopment is one of the most complex and fascinating aspects of human physiology that may be affected by early contact with gut microbiota (12–14, 35). Human brain development starts during the third gestational week and lasts through adolescence and into early adulthood in humans under the control of both genetic and environmental factors (36). The development of cognitive, emotional, and social brain circuits occur in parallel under the fine modulation by several molecular regulatory networks (37, 38). Critical windows in brain development have been described, during which neural circuits are particularly sensitive and even vulnerable to external factors, including gut microbiota composition (39, 40). Interestingly, early post-natal brain development overlap with gut microbiota establishment (33, 39, 40).

Animal models, in particular germ-free (GF) mice, have been crucial for the study of gut microbiota–brain axis in early phases of neurodevelopment (41). Seminal studies suggest that both the composition and the metabolic activity of gut microbiota at specific time points may influence HPA axis development (42) and have long-lasting impact on behavioral and neuroendocrine responses to stress (42–45). Gut microbiota may program the activity of multiple neurotransmitter systems in different brain regions inducing a long-term modulation of motor control and anxiety-like behavior in adult life (13, 35, 46). GF mice had a higher turnover rate of NA, DA, and serotonin 5-HT in the striatum compared with specific pathogen-free (SPF) mice (46). The serotonergic system seems to be particularly susceptible to early-life microbiota dynamics (47–50). Male GF animals showed a marked elevation in 5-HT and 5-hydroxyindoleacetic acid, its main metabolite, in the hippocampus compared with conventionally colonized control animals (48). Interestingly, post-weaning restoration of a normal flora failed to reverse the alterations in brain neurochemistry elicited by the lack of early life exposure to gut microbiota (48). Also, plasma 5-HT levels are affected by gut microbiota activity. In a metabolomics study, the colonization of GF mice induced a significant increase in plasma 5-HT (51), and bacterial metabolites were shown to stimulate 5-HT synthesis and secretion by enterochromaffin cells (20, 21). Intriguingly, the maternal separation in mice, an established model of early-life stress, induced profound changes in the gut microbiota that resulted in an anxiety-like phenotype (52).

The gut microbiota may also play a role in synapse maturation and synaptogenesis. In particular, GF animals when compared with SPF animals, showed higher striatal expression of synaptophysin and PSD 95, two markers of synaptogenesis and excitatory synapse maturation, respectively (46). Brain-derived neurotrophic factor (BDNF) is a key regulator of synaptic plasticity and neurogenesis in the brain and plays a crucial role in learning, memory, and mood regulation throughout life (53). In GF mice, *Bdnf* expression is significantly lower in the hippocampus, amygdala, and cingulate cortex compared with SPF mice (46). However, some inconsistency were reported about *Bdnf* expression in the hippocampus (42, 46, 48, 49).

Intriguingly, most of the reported neurodevelopmental alterations in GF mice occur differently in the two sexes (42, 46, 48, 49). Gut microbiota influence on neurogenesis is relevant for the normal gross morphology and ultrastructure of the amygdala and hippocampus (54, 55). While GF mice exhibit increased adult hippocampal neurogenesis in the dorsal hippocampus, subsequent post-weaning microbial colonization failed to reverse these changes, suggesting the existence of a critical developmental window in early life during which gut microbiota may program adult hippocampal neurogenesis (55). Gut microbiota may also be instrumental for the development of the blood–brain barrier (BBB). GF mice, starting from intrauterine life, displayed a life-long increased BBB permeability compared with mice with a normal gut flora that can partially be reverted by the exposure to pathogen-free gut microbiota during adult life (56).

Microglia, the macrophages that constitute the first-line immune defense of the CNS, play a central role in brain development, plasticity, and cognition and have been associated with the initiation or progression of several developmental and neurodegenerative diseases, including AD and PD (57, 58). Very recently, it was shown that microglia exhibited a time- and sex-specific susceptibility to gut microbiota depletion in mice (59). In particular, males seem to have their critical window during early *in utero* development, while females are more affected during adulthood. Microbiota alterations may have both acute and long-term effects on microglial functions. Remarkably, human fetal microglia showed significant similarities in the expression of key microglial genes when compared with murine counterparts (59). Finally, GF mice exhibited an increased myelination of neurons in the prefrontal cortex that could be reversed by colonization with a conventional microbiota following weaning (60).

Interventions on the early gut microbiota community (through the use of antibiotics, drastic changes in diet and/or pre/probiotic administration) may have profound effects on the gut–brain axis throughout life. For instance, antibiotic use during the first years of life was associated with neurocognitive outcomes later in life (e.g., depression, behavioral difficulties) (61).

In summary, several lines of evidence, although obtained mostly from animal models, suggest a relevant role played by the gut microbiota during the early phases of neurodevelopment, with possible long-standing effects later in life. The translatability of animal model findings to humans is obviously a priority but, also when ascertained, a comprehensive discussion should be started before implementing intervention strategies that could harm the mother–infant dyad in the first critical 1,000 days of life (62, 63).

## The Adult “Steady-State” Microbiota and CNS: Committing to a Stable Relationship

From birth till adulthood, bacterial diversity and functional capacity expand progressively, although at different rates across life stages (i.e., faster during infancy and slightly slower in early childhood) (64, 65).

In adulthood, gut microbial population fluctuates around a steady state (in terms of composition, diversity, and function) and remains quite resilient unless gross perturbations occur (e.g., major health conditions) (66). “Healthy” adult gut microbiota are consistently dominated by 2 main phyla (Bacteroidetes and Firmicutes), but more than 1,000 different bacterial species have been characterized and represent the vast human microbial collection (67–69). Each individual is characterized by a specific combination and proportion of different microbial species and subspecies (strains) that constitutes a unique microbial fingerprint (69). Despite this taxonomic inter-individual variability, adult gut microbiota display a relatively consistent functional capacity in healthy persons (70, 71). Importantly, microbial diversity and functional redundancy are positively associated with health, while decreased microbial richness and diversity and loss of functional redundancy characterize the microbiota in multiple disease conditions (66, 69, 72). Adult gut microbiota is influenced by several factors, including host genetics (73), nutrition and dietary habits (74, 75), xenobiotics (e.g., antibiotics) and other drugs (76–78), exercise (75, 79, 80), and circadian rhythm (81, 82).

Gut microbiota and brain dynamically interact also during adulthood. In adult mice, short-term oral administration of broad-spectrum antibiotics induced a decrease in anxiety and upregulated hippocampal expression of *Bdnf* (83). These changes were associated with a transient perturbation of microbiota but occurred independent of inflammatory status, vagal or sympathetic integrity, or alterations in gastrointestinal neurotransmitter levels (83). Adult neuroplasticity is sensitive to several environmental stimuli, including stress and gut microbiota alterations (84). Adult mice treated with antibiotics showed decreased hippocampal neurogenesis and memory retention (85). This effect was not completely rescued by the restoration of a normal flora by fecal transplant, unless supported by exercise or a probiotic cocktail administration (85).

Recent evidence suggests that complex microbiota-derived stimuli are requested for microglia maintenance also during adulthood (26, 59, 86). In particular, SCFAs, derived from bacterial fermentation processes, seem to regulate adult microglia homeostasis (26). Moreover, short-term antibiotic treatment in adult mice induce a rapid and sexually dimorphic (higher in females) change in microglial gene expression, reinforcing the concept that microbiota perturbations may have a relevant impact of microglia also during adulthood (59).

## The Second Brain Aging: Linking Gut Microbiota to Neurodegeneration

Aging is a process characterized by progressive functional decline of all physiological systems. In the gastrointestinal tract, aging involves the degeneration of enteric nervous system (ENS), alterations in gastrointestinal motility, perturbations in small intestinal permeability and mucosal defense system, which may promote the development of gastrointestinal diseases, affect the local and systemic inflammatory status, and deeply influence both the composition and function of resident microbiota (87–89).

Aging is also associated with broad changes in brain and whole body physiology that may influence gut microbiota–brain axis. In particular, the HPA axis is deeply perturbed, through a self-reinforcing cycle mediated by the hyperactivation of the HPA axis that leads to increased basal glucocorticoid release and the impaired HPA negative feedback due to reduced central glucocorticoid receptor expression (90, 91). HPA axis dysfunctions have been associated with decline in hippocampal volume and cognitive performance, and increased risk of late-life depression and anxiety (92, 93). Also circadian rhythm disruption, which is typical of aging, may be involved in this process, due to the potential effect on both cortisol level fluctuations and gut microbial activities (94, 95).

The aging brain is also deficient in the synthesis of neurotrophic factors, including BDNF (96) as well as several neurotransmitters, including 5-HT and DA, all of which lead to neuronal and cognitive dysfunction (97, 98). BBB breakdown is an early event in the aging human brain that begins in the hippocampus and may contribute to cognitive impairment (99).

Aging is also characterized by the progressive decline in immune function (immunosenescence) associated with a chronic, low-grade inflammation (inflamm-aging) (100, 101). Both processes may have many effects on the CNS, such as microglial activation, BBB breakdown, and increase in oxidative damage that may contribute to neurodegenerative and neuropsychiatric diseases (100). Remarkably, recent data suggest that, in old mice, gut microbiota contribute to inflamm-aging, and that this inflammatory phenotype may be transferred to young GF mice (102).

Major taxonomic shifts and a consistent decrease in microbial richness and diversity have been reported in people 65 years of age and older and these changes were associated with worsening of health status and frailty (89, 103). Similar findings were also obtained in mice (104).

The characterization of gut microbiota of centenarians revealed the presence of significant compositional differences across life stages till extreme ages (105). In particular, core microbiota (mostly composed by the members of Ruminococcaceae, Lachnospiraceae, and Bacteroidaceae families) seem to accompany human life, decreasing in abundance along with aging (105). In longevity and extreme longevity, an enrichment in some subdominant health-associated groups (e.g., *Akkermansia, Bifidobacterium*, and *Christensenellaceae*) occurs, even with the support of some opportunistic and allochthonous bacteria (105).

Recently, the effects of aging on the microbiota gut–brain axis were assessed in male mice (106). Aged mice showed significant shifts in gut microbiota that were associated with deficits in spatial memory and increases in anxiety-like behaviors compared with young adult mice (106). These changes were positively correlated with the abundance of bacteria from the Porphyromonadaceae family. Aged mice also exhibited increased gut permeability that was associated with elevations in peripheral pro-inflammatory cytokines (106).

These preliminary findings suggest that age-related changes in gut microbiota may impact behavioral and cognitive functions and support the relevance of the alteration in gut permeability and peripheral inflammation in mediating these effects.

As outlined earlier, the possible link between early gut microbiota–brain interactions and late onset neurological conditions, including AD and PD, is an intriguing area of research (15).

### Alzheimer’s Disease

**Figure 2:**
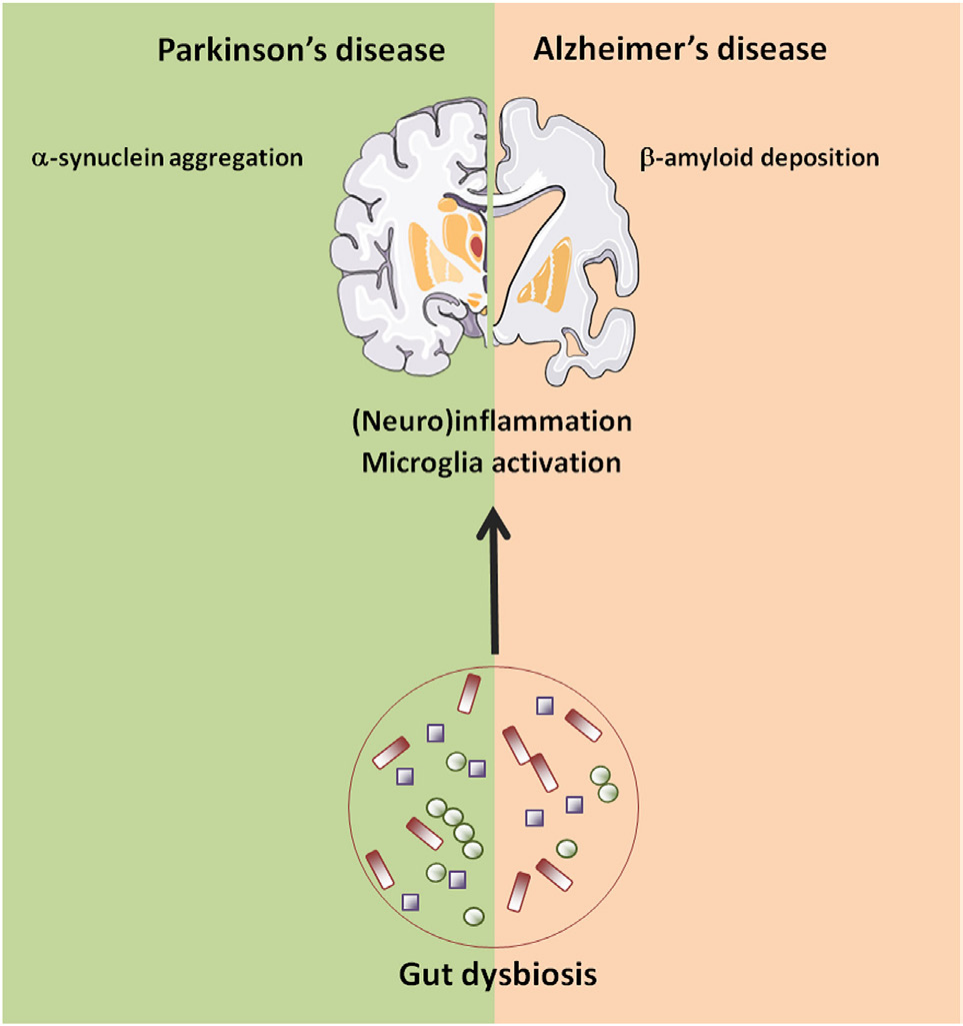
Age-related changes in gut–brain axis possibly involved in neurodegeneration. Abbreviations: AD, Alzheimer’s disease; PD, Parkinson’s disease.

In AD, the most common form of age-related dementia, deposition of protein aggregates composed of amyloid-β (Aβ) peptide and tau in brain tissues impairs cognitive function (107). Both host- and environmental factors that regulate these processes have been described, including a potential role for gut microbiota (108) (Figure 2).

In AD, reduced microbial richness and diversity were observed, with low abundance of Firmicutes and *Bifidobacterium* and increased Bacteroidetes that characterized the microbiome of AD patients (108). Correlations were found between the levels of *Bacteroides, Turicibacter*, and *SMB53* and the concentration of glial activation biomarkers in cerebrospinal fluid of AD (108).

An increase in the abundance of the pro-inflammatory *Escherichia*/*Shigella* taxon, and a corresponding reduction in the anti-inflammatory *E. rectale* was associated with higher levels of inflammatory mediators in patients with cognitive impairment and brain amyloidosis (109). Also in a mouse model overexpressing amyloid precursor protein and presenilin 1 (APPPS1), a distinct microbial signature was observed with an increase in *Rikenellaceae* and decreased *Allobaculum* and *Akkermansia* compared with age-matched wild-type controls (110).

Interestingly, reduced levels of *Akkermansia* characterize gut microbiota of mice with obesity and type 2 diabetes (111), two potentially modifiable risk factors for AD (107). Importantly, both young and old GF APPPS1 transgenic mice displayed a drastic reduction of cerebral Aβ pathology when compared with control mice, along with a reduced microgliosis (110). Further to this, colonization of GF-APPPS1 transgenic mice with microbiota from conventionally raised APPPS1 transgenic mice increased cerebral Aβ pathology, while colonization with microbiota from wild-type mice was less effective in increasing cerebral Aβ levels (110). Notably, GF-APPPS1 displayed increased levels of the Aβ-degrading enzymes insulin degrading enzyme and neprilysin degrading enzyme, suggesting a mechanism through which gut microbiota influence cerebral Aβ amyloidosis (110).

In the same mouse model of AD, life-long antibiotic treatment induced a considerable perturbation in gut microbial composition (including an expansion of *Akkermansia*) that was associated with marked changes in the circulating cytokine/chemokine network, a striking reduction in amyloid plaque deposition, and a concomitant increase in soluble Aβ (112). This was accompanied by alterations in neuroinflammatory milieu that lead to reduced plaque-localized gliosis and altered microglial morphology (112). Remarkably, early post-natal antibiotic treatment alone resulted in long-term alterations in gut microbial genera that were associated with changes in the inflammatory environment of serum and cerebrospinal fluid and attenuated Aβ amyloidosis in a manner similar to that observed in mice subjected to life-long antibiotic selection pressure (113). These findings corroborate the hypothesis of the presence of critical developmental periods in which the commensal microbiota manipulation may have long-lasting effects on host immunity and potential implications for neurodegenerative diseases.

In another model of AD, the 5xFAD transgenic mouse, elevated levels of APP were found not only in the brain but also in the different gut districts and this was associated with a distinct fecal microbiota profile relative to wild-type animals, with an increase in pro-inflammatory species (e.g., *Clostridium leptum*) (114).

Alterations in gut microbiota composition together with the increase in intestinal permeability with age may lead to the translocation of microbes or microbial components [i.e., lipopolysaccharide (LPS)] from the gut to induce systemic and CNS inflammation (115). Interestingly, *in vitro* and *in vivo* studies have demonstrated a possible association between LPS and AD pathology. Coincubation of Aβ peptide with LPS potentiated amyloid fibril formation (116), and systemic administration of LPS in wild-type and transgenic AD mice induced neuroinflammation, amyloid deposition, and tau pathology (117–119). Moreover, in postmortem brain parenchyma and blood vessels from patients with AD, levels of LPS and Gram-negative *E*. *coli* fragments were greater compared with control brains and colocalized with amyloid plaques (120).

While the study of the microbiota gut–brain axis in AD is still in its infancy, promising preclinical data suggest that the modulation of gut microbiota through dietary ingredients or probiotics may provide a means to counteract the development or progression of neurodegenerative disease. For instance, in a triple-transgenic mouse model of AD (3xTg-AD), a formulation of lactic acid bacteria and bifidobacteria changed the composition of gut microbiota, stimulated the production of beneficial metabolites (e.g., increased SCFAs), reduced the levels of pro-inflammatory cytokines, increased gut hormones concentration and positively modulate quality control processes and proteolysis, reducing Aβ load and improving cognitive function (121). Moreover, the administration of the probiotic mixture VSL#3 to aged rats induced a robust perturbation in gut microbiota composition, that was accompanied by gene expression changes in the brain cortex, attenuated age-related deficits in long-term potentiation, decreased microglial activation, and increased BDNF and synapsin levels (122). In addition, 3-hydroxybenzoic acid and 3-(3-hydroxyphenyl)propionic acid, the phenolic products of microbial conversion of grape seed polyphenol extracts (and other dietary polyphenols), may potently interfere with the assembly of Aβ peptides into neurotoxic Aβ aggregates *in vitro* (123).

Despite these interesting preliminary findings, more work is needed to determine whether gut microbiota modulation may be employed for the prevention and/or treatment of AD pathogenic processes.

### Parkinson’s Disease

Parkinson’s disease is the second most common neurodegenerative disorder, affecting 2–3% of the population ≥65 years of age (124, 125). Degeneration of the dopaminergic nigro-striatal pathway and widespread intracellular α-synuclein accumulation are the neuropathological hallmarks of PD that are associated with bradykinesia and other cardinal motor and non-motor features (126).

Gastrointestinal dysfunction, in particular in the form of constipation, is among the most frequent prodromal non-motor symptoms of PD that may precede motor symptoms by decades (126). At later disease stages, oral issues including drooling and swallowing problems and delays in gastric emptying further exacerbate gastrointestinal dysfunction (127). Aggregates of α-synuclein have been retrieved in the mucosal and submucosal nerve fibers and ganglia of the ENSs of PD patients at early disease stages (128, 129). In addition, some observations from experimental models support the intriguing hypothesis that intestinal α-synuclein may spread to the brain *via* postganglionic enteric neurons and the vagus nerve (130). Interestingly, the risk of developing PD was significantly decreased in patients who underwent a full truncal vagotomy compared with those who underwent selective vagotomy and in the general population (131).

Not surprisingly, gastrointestinal disturbances in people with PD are accompanied by alterations in fecal and mucosal microbial populations (31, 132–134). In particular, a reduced abundance of Prevotellaceae, mucin producers that regulate intestinal permeability, was commonly reported in PD patients (31, 132, 135, 136), while *Enterobacteriaceae* were positively associated with the severity of postural instability and gait difficulty (31). *Clostridium coccoides* group was high in early PD patients, while *Lactobacillus gasseri* subgroup was high in advanced PD patients (132). A pro-inflammatory dysbiosis, characterized by low counts of “anti-inflammatory” butyrate-producing bacteria from the genera *Blautia, Coprococcus*, and *Roseburia* and higher “pro-inflammatory” Proteobacteria of the genus *Ralstonia* was also reported in individual with PD (133). Individuals affected by PD also showed lower levels of SCFA concentrations, derived from host–microbiota cometabolism, that may have neuroactive and immunomodulating properties (135). Other evidence of microbiota dysregulation in PD includes small intestine bacterial overgrowth and high rates of *Helicobacter pylori* infection (137, 138). It is worth noting that this infection has also been involved in the pathogenesis of AD (139). Finally, the total abundance of intestinal bacterial was found to decrease during PD progression, with a low count of *Bifidobacterium* associated with worsening of PD symptoms (134).

Collectively, these findings suggest that perturbations in gut microbiota structure and function may be associated with the development and progression of PD through several potential mechanisms, including inflammation and bacterial translocation (Figure 2). However, findings in humans remain largely descriptive. Again, animal models provided some useful insights into the physiopathological mechanisms linking gut dysbiosis to PD. Under GF conditions, or when bacteria were depleted in post-natal life following antibiotic treatment, transgenic mice overexpressing α-synuclein showed reduced microglia activation, α-synuclein inclusions, gastrointestinal symptoms, and motor deficits compared with animals with a complex microbiota (140). Moreover, administration of a mixture of microbially derived SCFAs (acetate, propionate, and butyrate) restored all major features of PD in GF mice, suggesting that microbial metabolic mediators may promote microglia activation and α-synuclein aggregation and contribute to motor dysfunction in PD (140). Remarkably, mice transplanted with PD microbiota compared with mice who received microbiota from healthy human controls displayed enhanced motor dysfunction, suggesting that dysbiosis may be the environmental factor that combined with a genetic predisposition (α-synuclein overexpression) influences disease outcomes in mice (140).

As already outlined for AD, in neurodegenerative diseases, including PD, the passage of bacterial products from the intestine to the circulation and into brain, or “molecular mimicry” processes induced by bacterial amyloids may trigger a persistent neuroinflammation (28, 141, 142) that in turn contributes to neuronal dysfunction and death (143). In this scenario, it has recently been proposed that Aβ production and aggregation may originally act as an antimicrobial defense and then infectious or sterile inflammatory stimuli may drive amyloidosis (144).

While it is currently recommended the use of fermented milk containing probiotics and prebiotic fiber in PD patients with constipation (145), the possible beneficial effects of the manipulation of gut microbiota (through diet, live bacteria, or microbiota transplantation) on the initiation or progression of the neurodegenerative process have not yet been explored. Further studies are also needed to assess the possible interactions among these interventions and levodopa uptake and availability.

## Concluding Remarks

At the beginning of the twentieth century, the Nobel Prize winner Elie Metchnikoff theorized in his tracts, *The Nature of Man: Studies in Optimistic Philosophy* (1903) and *The Prolongation of Life: Optimistic Studies* (1907), that health status could be improved and senility delayed by replacing the native gut microbes with lactic acid bacteria such as those present in yogurt (146). In the past few decades, this idea was resumed and updated under the influence of methodological and technological advances in science (147). A more ecological perspective was then embraced and the concepts of complexity, (dis)harmony, (Nash)equilibrium, and personalization/precision were introduced to capture the dynamic aspects of gut microbiota–host relationship (66, 147–149).

**Figure 3:**
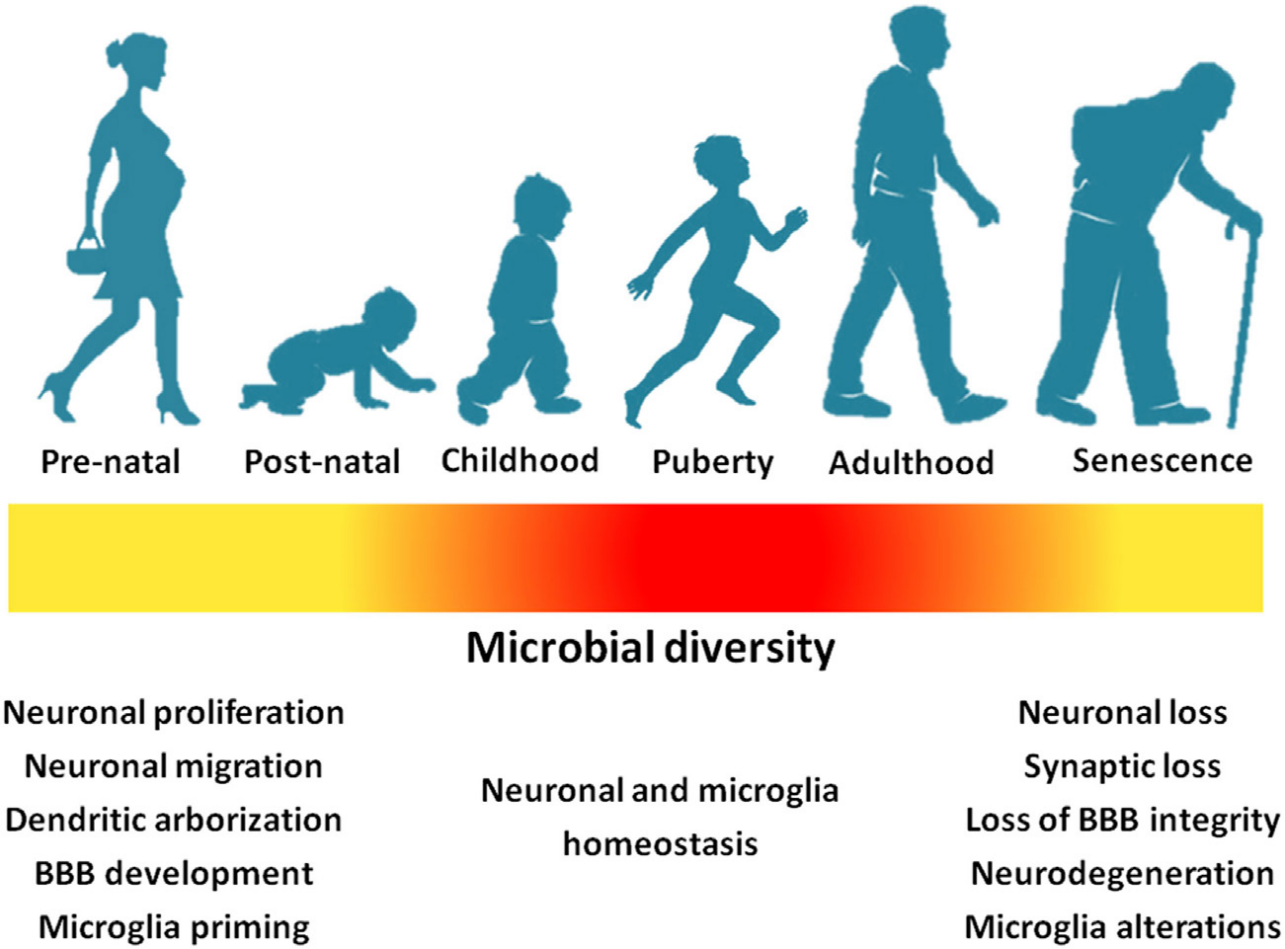
Gut microbial richness and diversity across life stages impact neurodevelopment and the central nervous homeostasis (yellow: low richness/diversity; red: high richness/diversity). Abbreviation: BBB, blood–brain barrier.

While the study of microbiota gut–brain axis is still in infancy, a number of potential mechanisms (and, hence, plausible targets) have begun to be unveiled. Early-life interactions between host and colonizing gut microbes seem to influence the way in which the nervous system starts obtaining information about the external and internal environment in critical phases of neurodevelopment. BBB establishment and function, central inflammatory processes and neurogenesis may be differentially affected by the gut microbial assemblies and their metabolic products (Figure 3). Evidence is also accumulating for a role of life-long microbiota–host interactions in age-related disorders such as AD and PD.

Taken together, these data open up the possibility of developing interventions targeting the gut microbiota (in particular at the extreme ages of life) to improve brain health. Preclinical studies have suggested the efficacy of the modulation of the gut microbiota in ameliorating conditions such as depression and neurodegenerative diseases (150). A new term, “psychobiotics” (and related “psychobiotic properties”), was coined to define live bacteria (probiotics) and nutritional support for such bacteria (prebiotics), but also virtually any exogenous factor, such as diet, exercise, and drugs, acting on brain through bacterially mediated effects (19).

Despite the “optimistic nature” of this 100-year-old idea, future research should tackle several challenging questions before truly effective interventions in humans may be implemented. For instance, most of the published studies have only associated the gut microbiota with diseases without proving any causation (1, 151). It is therefore crucial to assess whether changes in microbiota underpin disease pathophysiology or are just epiphenomena. Further to this, the microbial properties that are necessary to support proper neurodevelopment and prevent neurodegeneration should be clearly established. In addition, sufficiently powered, rigorous clinical trials should be conducted to assess the translatability of animal model findings to human conditions.

## Author Contributions

RC, EM, and AP conceived the manuscript. RC, MRLM, and AP drafted the paper. RB, FL, and EM supervised and edited the manuscript. All authors read and approved the final version of the paper.

## Conflict of Interest Statement

The authors declare that the research was conducted in the absence of any commercial or financial relationships that could be construed as a potential conflict of interest.
